# Neutralizing autoantibodies against interferon alpha in systemic lupus erythematosus: Prevalence, age of onset, and clinical associations

**DOI:** 10.1177/09612033261432154

**Published:** 2026-03-06

**Authors:** Elsa Grenmyr, Birgitta Gullstrand, Andreas Jern, Niklas Björklund, Robin Kahn, Fredrik Kahn, Petrus Linge, Andreas Jönsen, Anders A. Bengtsson

**Affiliations:** 1 Department of Clinical Sciences, Rheumatology, 156327 Lund University and Skåne University Hospital, Lund, Sweden; 2 Department of Clinical Sciences, Pediatrics and Wallenberg Centre for Molecular Medicine, 156327 Lund University and Skåne University Hospital, Lund, Sweden; 3 Department of Clinical Sciences, Infection Medicine and Wallenberg Centre for Molecular Medicine, 156327 Lund University and Skåne University Hospital, Lund, Sweden

**Keywords:** Systemic lupus erythematosus, neutralizing interferon autoantibodies, organ damage, disease phenotypes, interferon

## Abstract

**Objective:**

Type I interferons (IFN) drive systemic lupus erythematosus (SLE) pathogenesis. Some patients develop neutralizing IFN autoantibodies (anti-IFN ab), which theoretically could modify disease activity. We aimed to determine the prevalence of anti-IFN ab in patients with SLE, identify the age and when during the disease course of anti-IFN ab emerge, and assess their association with organ damage.

**Methods:**

This cross-sectional study included 173 SLE patients from the Lund Lupus Cohort. Samples taken at routine outpatient visits were analyzed for anti-IFN ab using ELISA, and positive samples were tested for IFN neutralizing capacity with a gene-reporter assay. Longitudinal samples were analyzed to determine the time-point and age of first positive sample. Demographic and clinical data were obtained from research registries.

**Results:**

Eighteen (10.4%) patients were positive for anti-IFN ab by ELISA. Among these, antibodies from nine patients (5.2%) displayed IFN neutralizing capacity. No statistically significant differences were detected between patients positive for neutralizing antibodies and antibody-negative patients with respect to demography, organ damage or ACR classification criteria. The group with neutralizing antibodies were slightly older (median age 59 vs 45 years, p = .14) and had a higher proportion of renal involvement (67% vs 33%, p = .088). Longitudinal analysis of samples from patients with neutralizing anti-IFN ab revealed two age-related patterns: late-onset (≥65 years, *n* = 4), including one patient positive at diagnosis at age 69, and early-onset (≤40 years, *n* = 5), with antibodies present at or soon after diagnosis in four cases. Organ damage did not differ between patients with or without neutralizing antibodies (p = .65). At the latest follow-up (2–38 years after anti-IFN ab detection), three of nine patients remained free of organ damage.

**Conclusions:**

Approximately 5% of SLE patients have neutralizing anti-IFN antibodies, which may present early in disease or develop later in life. While late-onset antibodies may reflect age-related changes in immune regulation and early-onset antibodies could potentially modulate IFN-driven mechanisms, our data do not support a protective effect against organ damage.

## Introduction

Type I interferons (IFNs) play a central role in antiviral immunity and in the pathogenesis of systemic lupus erythematosus (SLE). Type I IFNs are linked to SLE pathogenesis, disease activity and disease severity in patients with SLE.^1^ Patients with type I IFN signature more often present with earlier disease onset and renal involvement and are more frequently treated with glucocorticoids and immunosuppressants to control disease activity.^2^ SLE is characterized by a loss of self-tolerance and development of a broad range of autoantibodies.^3^ Many of these autoantibodies target intracellular antigens, including DNA and RNA. Nucleic acids containing immune complexes activate innate immune receptors such as TLR7 and TLR9, stimulating the production of type I IFNs and other pro-inflammatory cytokines. This response amplifies immune activation and IFN-driven autoimmunity where type I IFN activate antigen-presenting cells, enhancing B-cell survival, plasma cell differentiation and expansion of autoreactive T-cells.^3^

During the SARS-CoV2 pandemic, neutralizing autoantibodies against type I IFNs (anti-IFN ab) emerged as a significant risk factor for severe COVID-19 outcome and death.^4^ Historically, these autoantibodies were primarily recognized in autoimmune polyendocrinopathy syndrome type I and thymoma, and other diseases caused by genetic or acquired defects of T-cell tolerance.^5^ Anti-IFN ab was previously regarded as having limited clinical relevance in viral pathogenesis. However, studies of COVID-19 revealed that up to 20% of adults who died with the infection harbored anti-IFN ab. In the general population under 70 years of age, their prevalence is estimated at 0.5–1%, but this increases sharply to 2-6% in individuals over age 70 ^6^ . These autoantibodies were also shown to increase the risk of severe disease in other viral infections, including influenza and West Nile Virus, and may also pose a risk of complications following vaccination with live attenuated yellow fever virus vaccine.^7–9^

In the context of SLE, where IFN pathways are central to disease pathology, neutralizing anti-IFN ab might have a protective effect by dampening IFN-driven immune activation. Early studies reported 10-25% of patients with SLE being positive for anti-IFN ab, but with conflicting results of their clinical relevance.^10,11^ More recent investigations reported a prevalence of 3-5% and association with reduced disease activity.^12,13^ Whether anti-IFN ab provide protection against organ damage is less clear.

In the present investigation we sought to (1) determine the prevalence of anti-IFN ab in a Swedish SLE cohort, (2) investigate at what age and time during the disease course anti-IFN ab appear, and (3) assess their association with organ damage.

## Methods

### Study population

This cross-sectional study included 173 prevalent patients with SLE from the Lund Lupus Cohort, attending routine outpatient visits at the Department of Rheumatology, Skåne University Hospital, Lund, Sweden. Samples were retrieved from the cohort biobank. Cross-sectional samples were screened for anti-IFN ab using an in-house ELISA. To establish the cut-off for anti-IFN ab positivity in the ELISA, samples from 59 normal healthy individuals and 51 non-SLE disease controls (15 systemic sclerosis, 18 rheumatoid arthritis, 18 with cardiovascular disease [without rheumatic disease]) were also analyzed.

### Longitudinal analysis and clinical data

For patients with neutralizing anti-IFN ab in the cross-sectional study, retrospective samples (4-10 samples per patient) were analyzed by ELISA to determine the timepoint of anti-IFN development. Samples to analyze were chosen with following strategy: a) one sample taken as close to disease onset as possible, b) samples with 1 to 6 years interval between diagnosis and cross-sectional time-point, and c) one sample taken after the cross-sectional time-point (0-10 years after cross-section). Demographic data, classification according to American College of Rheumatology (ACR) classification criteria 1982, disease activity according SLE Disease Activity Index 2000 (SLEDAI) and organ damage according to the Systemic Lupus International Collaborating Clinics Damage Index (SDI) were retrieved from the SLE research registers at the Department of Rheumatology, Skåne University Hospital, Lund, Sweden.

### ELISA

Microtiter plates (Sarstedt, 82.1581.200) were coated overnight with recombinant human IFN-α2a (Novus Biologicals, NBP2-34971). Plates were blocked with 5% dry milk in PBS containing 0.05% Tween 20 (PBS-T). Wells were washed three times with PBS-T between steps. Serum samples, diluted 1:100 in blocking buffer, were added and incubated for 2h, followed by alkaline phosphatase conjugated anti-human-IgG antibody (Sigma Aldrich, A3312). The substrate, disodium-p-nitrophenyl phosphate (Sigma Aldrich, S0942; 1 mg/ml dissolved in 10% [w/v] diethanolamine pH 9.8 containing 50 mM MgCl_2_) was added, and the absorbance was measured at 405 nm. Background values (from uncoated wells) were subtracted from duplicate means and arbitrary anti-IFN concentrations were calculated from titration curves obtained from Rontalizumab (Invitrogen, MA5-41908). The 95th percentile of values from the 54 healthy subjects and 51 disease controls was used as the cut-off for positivity.

### Neutralization assay

All anti-IFN ab positive SLE samples in the cross-sectional study (*n* = 18) were further tested using a cell-based reporter gene assay. Reporter THP1-Dual™ Cells (Invivogen, thpd-nfis) were cultured in RPMI-1640 Medium (Gibco A1049101) supplemented with 10% heat-inactivated fetal calf serum (FCS), 100 U/ml penicillin, 0.1 mg/ml streptomycin and 0.1 mg/ml Normocin at 37°C in a humidified 5% CO_2_ incubator. To maintain selection pressure, 10 μg/ml of Blasticidin and 100 μg/ml of Zeocin were added. Samples diluted 1:10 in RPMI-1640+10% FCS and recombinant human IFN-α2a were incubated for 30 minutes, while shaking, at room temperature before addition to reporter cells (0.5*10^6^ cells/ml). Cells and samples were incubated for 24h at 37°C/5% CO_2_. Supernatant (20 μL) was mixed with 50 μL QUANTI-Luc™4 Lucia/Gaussia (Invivogen, rep-qcl4lg) in a white plate (Thermo Scientific, 236105), and IFN-induced luminescence was measured with a luminometer. Samples inhibiting ≥70% of IFN-α2a induced signal were characterized as neutralizing.

### Statistical tests

Age at the study, age at diagnosis, disease duration, SDI and ACR classification criteria were compared between the group with neutralizing anti-IFN ab group (*n* = 9) and the patient group negative for anti-IFN ab (*n* = 155) using the Mann–Whitney U test for continuous variables and Chi-squared test for categorical variables. RStudio 2025.09.0 and GraphPad Prism 10.5.0, were used for tables, figures and statistical calculations.

## Results

We detected anti-IFN antibodies by ELISA in 18 (10.4%) of the 173 analysed patients with SLE, in 1 of 59 of normal healthy subjects and 4 of 51 disease controls (Supplemental Figure S1). When further analysing the neutralizing capacity of the ELISA-positive samples using the cell-based assay, 9 samples (5.2%) from patients with SLE were able to inhibit IFN-induced luciferase expression (Supplemental Figure S2).

The cohort consisted of 89.5 % women, the median age was 45 years (interquartile range [IQR] 33–61) and median age at diagnosis 33 years (IQR 22–44) (Table 1). Median disease duration was 11 years (IQR 4–19).

**Table 1.** Demographic and clinical characteristics of the cross-sectional cohort.

Characteristic	Overall *N* = 173^a^	Anti-IFN autoantibodies			*p*-value^b^
		Neutralizing *N* = 9^a^	Non neutralizing *N* = 9^a^	Negative *N* = 155^a^	
Females	155 (90%)	8 (89%)	8 (89%)	139 (90%)	
Age at study	45 (33-60) [18-81]	59 (45-72) [26-73]	44 (38-65) [24-67]	44 (33-59) [18-81]	0.14
Age at diagnosis	33 (22-43) [8-75]	30 (23-45) [19-69]	41 (26-44) [22-62]	33 (21-43) [8-75]	0.91
Disease duration	11 (4-19) [0-46]	25 (3-28) [2-46]	8 (5-14) [2-21]	11 (4-19) [0-45]	0.20
SLICC/ACR damage index	1 (0-2) [0-9]	1 (0-3) [0-8]	0 (0-2) [0-3]	1 (0-2) [0-9]	0.65
No. of ACR criteria met	6 (5-7) [3-10]	5 (5-7) [5-8]	6 (6-7) [4-8]	5 (5-7) [3-10]	0.68
Malar rash	97 (56%)	4 (44%)	6 (67%)	87 (56%)	0.73
Discoid rash	41 (24%)	0 (0%)	2 (22%)	39 (25%)	0.19
Photosensitivity	114 (66%)	4 (44%)	7 (78%)	103 (66%)	0.32
Oral ulcers	55 (32%)	3 (33%)	4 (44%)	48 (31%)	>0.99
Arthritis	141 (82%)	8 (89%)	9 (100%)	124 (80%)	0.82
Serositis	72 (42%)	4 (44%)	4 (44%)	64 (41%)	>0.99
Neurological disorders	13 (7.5%)	1 (11%)	1 (11%)	11 (7.1%)	>0.99
Renal disorders	60 (35%)	6 (67%)	3 (33%)	51 (33%)	0.088
Hematologic disorder	122 (71%)	6 (67%)	6 (67%)	110 (71%)	>0.99
Immunologic disorder	124 (72%)	9 (100%)	7 (78%)	108 (70%)	0.11
Anti-nuclear antibody	170 (98%)	9 (100%)	8 (89%)	153 (99%)	>0.99

^a^n (%); Median (Q1-Q3) [Min-Max].

^b^Comparing neutralizing vs negative group, Mann-Whitney test for continuous; Chi-squared test for categorical.

Abbreviations: SLE, systemic lupus erythematosus; ACR, American College of Rheumatology, SDI, Systemic Lupus International Collaborating Clinics Damage Index, IFN, interferon

No statistically significant differences were observed between patients with neutralizing antibodies and antibody-negative patients, in terms of age at cross-section, age at diagnosis, disease duration, organ damage or number of ACR classification criteria. Nevertheless, some tendencies indicating possible differences in SLE phenotypes between these two groups were observed. Patients with neutralizing anti-IFN ab were generally older at the study (median age 59 vs 45 years, p = .14). According to the ACR classification criteria, they more often had renal disease (67 vs 33%, p = .088), none had discoid rash (0 vs 25%, p = .19), and all fulfilled the immunological disorder criterion (100 vs 71%, p = .11). Organ damage, assessed by SDI, were not different between patients with neutralizing anti-IFN ab and anti-IFN ab negative patients (Table 1).

**Figure 1.**
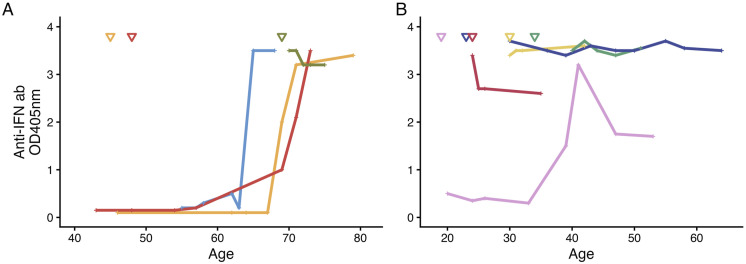
Longitudinal samples from patients with neutralizing anti-IFN antibodies, analyzed with ELISA. A) Late-onset anti-IFN. >65 years old, n=4. B) Early-onset anti-IFN. <40 years old, n=5. ▽=Years of age at diagnosis time-point. Abbreviations: SLE, systemic lupus erythematosus; IFN, interferon; ab, antibodies; OD, optical density

Analysis of longitudinal samples from patients with neutralizing anti-IFN ab revealed two distinct patterns, depicted in Figure 1. Late-onset of neutralizing anti-IFN antibodies, at age 65 or older (Figure 1(a)), or early-onset, at age 40 or younger (Figure 1(b)).

Late-onset of neutralizing antibodies were found in four patients. Three of these patients were anti-IFN ab negative for 23-44 years after their SLE diagnosis, before development of anti-IFN ab. The fourth patient, a male, was positive for neutralizing anti-IFN ab at SLE diagnosis at age 69 (Figure 1(a), Table 2).

**Table 2.** Clinical characteristics of patients with neutralizing anti-IFN antibodies

SLE diagnosis			1st anti-IFN ab			10-year SDI	Latest follow-up				
Sex	Age	Year	Age	Disease duration	SDI		Age	Disease duration	ACR	SDI	Type of damage
Late onset											
Female	21	1965	65	44	3	0	79	58	8	5	Cardiovascular, Musculoskeletal, Skin
Female	45	1983	70	25	0	0	80	35	7	0	None
Female	48	1986	71	23	5	4	73	25	5	8	Ocular, Neuropsychiatric, cardiovascular, diabetes
Male	69	2008	69	0	0	6	81	11	5	6	Renal, cardiovascular, peripheral vascular, Musculoskeletal
Early onset											
Female	23	1974	30	7	2	3	68	45	7	4	Ocular, pulmonary, Musculoskeletal, Malignancy
Female	19	1988	39	20	0	0	56	36	5	1	Ocular
Female	34	1999	40	6	0	1	59	25	6	3	Renal, gastrointestinal
Female	24	2011	24	0	0	0	37	13	5	0	None
Female	30	2011	30	0	0	0	43	13	5	0	None

Abbreviations: SLE, systemic lupus erythematosus; IFN, interferon; ACR, American College of Rheumatology classification criteria; SDI, Systemic Lupus International Collaborating Clinics Damage Index.

Early-onset of neutralizing antibodies were found in five patients. In four cases, antibodies were present at diagnosis of SLE or in the earliest available sample within seven years of disease duration. In the fifth patient, neutralizing anti-IFN ab were detected at age 39 after 19 years of disease (Figure 1(B), Table 2).

Six of the nine patients with neutralizing anti-IFN had SDI = 0 at the timepoint for the 1^st^ anti-IFN ab positive sample, which occurred at 0-44 years after SLE diagnosis. At the latest follow-up, 2-38 years after the 1^st^ anti-IFN ab positive sample, three of the nine patients remained free of organ damage (Table 2). Patients with neutralizing anti-IFN were not protected from organ damage nor from ongoing disease activity, assessed using SLEDAI (Supplemental Figure S3A-D).

## Discussion

This study aimed to investigate the prevalence of neutralizing anti-IFN antibodies in patients with SLE, and evaluate their association with organ damage, as anti-IFN may have a protective effect in the SLE pathogenesis. Our study confirms that approximately 5% of SLE patients carry neutralizing anti-IFN, consistent with previously published data.^12,13^ The presence of these antibodies raises questions about their biological and clinical consequences in SLE.

A central finding in our investigation was the identification of two distinct age-related patterns of anti-IFN ab development. In one subset of patients, we observed late onset (≥65 years of age) of anti-IFN ab, comparable to the general population seen in the large population studies.^6^ Late-onset of anti-IFN ab may be unrelated to SLE, and more likely reflects immunosenescence and age-associated autoantibody development.^14^

The other subset of patients, early-onset (≤40 years of age) of anti-IFN ab, may emerge due to different mechanisms compared with late-onset. Early-onset of anti-IFN ab may arise from lupus-associated mechanisms of autoantibody production, or from impaired thymic function, similar to what has been described in other conditions where anti-IFN ab occur.^5^ It is reasonable to hypothesize that early development of neutralizing anti-IFN ab may have potential to modulate IFN-driven disease activity in SLE, in analogy to the effects of pharmacological treatment targeting type I IFN system. Our results do not support that neutralizing anti-IFN ab could be protective with respect to organ damage.

This study is limited by its small sample size, and the potential protective effects of these antibodies therefore remain unclear. Analysis of subsets of organ damage, or differences between late- and early-onset of anti-IFN ab was not feasible to analyse, due to the sample size. Furthermore, the SDI includes all organ damage occurring after diagnosis, regardless of cause, and is not restricted to SLE-related damage.

### Conclusion

This study demonstrates that approximately 5% of SLE patients harbour neutralizing autoantibodies against IFN-α. These antibodies emerge along two distinct age patterns, either early in the disease course (<40 years) or late in life (>65 years). While early-onset antibodies may attenuate IFN-driven mechanisms, their presence does not consistently prevent organ damage. Late-onset antibodies likely reflect age-related immune changes rather than lupus-specific processes. However, as these observations are based on a limited number of individuals, the findings should be interpreted with caution and cannot be generalized. Nevertheless, they open a possibility for future longitudinal investigations in larger cohorts to further explore the clinical implications of anti-IFN antibodies in SLE.

## Supplemental material

Supplemental material - Neutralizing autoantibodies against interferon alpha in systemic lupus erythematosus: Prevalence, age of onset, and clinical associations Supplemental material for Neutralizing autoantibodies against interferon alpha in systemic lupus erythematosus: Prevalence, age of onset, and clinical associations by Elsa Grenmyr, Birgitta Gullstrand, Andreas Jern, Niklas Björklund, Robin Kahn, Fredrik Kahn, Petrus Linge, Andreas Jönsen, Anders A Bengtsson in Lupus.

## Data Availability

The data that support the findings of this study are available from the corresponding author upon reasonable request.*

## Appendix

### Abbreviations

ACR: American College of Rheumatology
Anti-IFN ab: autoantibodies against type I IFNs
IFN: interferon
IQR: interquartile range
SLE: systemic lupus erythematosus
SDI: Systemic Lupus International Collaborating Clinics Damage Index
