# Supplementary material for: Neutralizing autoantibodies against interferon alpha in systemic lupus erythematosus: Prevalence, age of onset, and clinical associations
Source: Lupus. 2026 Mar 6;35(6):623–9. doi: 10.1177/09612033261432154 (PMC13047216; doi:10.1177/09612033261432154)
Supplement: Supplemental material - Neutralizing autoantibodies against interferon alpha in systemic lupus erythematosus: Prevalence, age of onset, and clinical associations [file sj-pdf-1-lup-10.1177_09612033261432154.pdf]

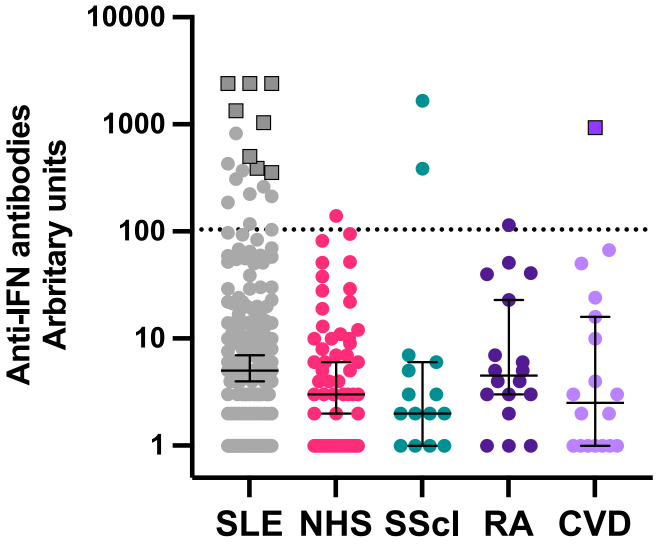

Supplementary Figure S1. ELISA results. SLE patients n=173, NHS, normal healthy subjects n=59, SScl, systemic sclerosis n=15, RA, rheumatoid arthritis n=18, cardiovascular disease, non-rheumatic n=15. Squares represent IFN neutralizing samples.

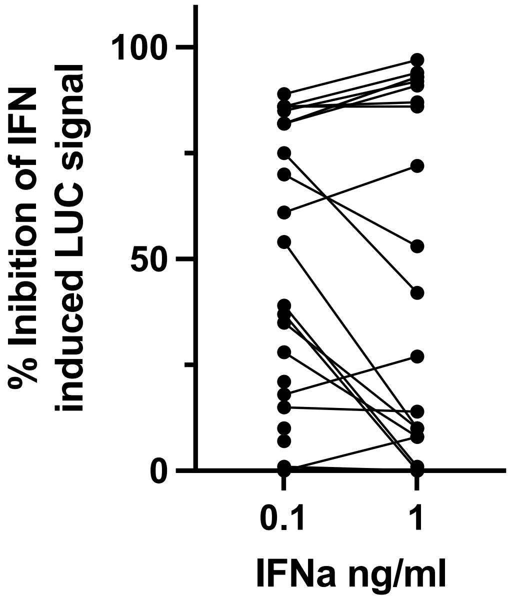

Supplementary Figure S2. Neutralization results. Anti-IFN positive samples tested for inhibition of IFNa induced luciferase (LUC) signaling. Percent inhibition was calculated against an anti-IFN negative sample.

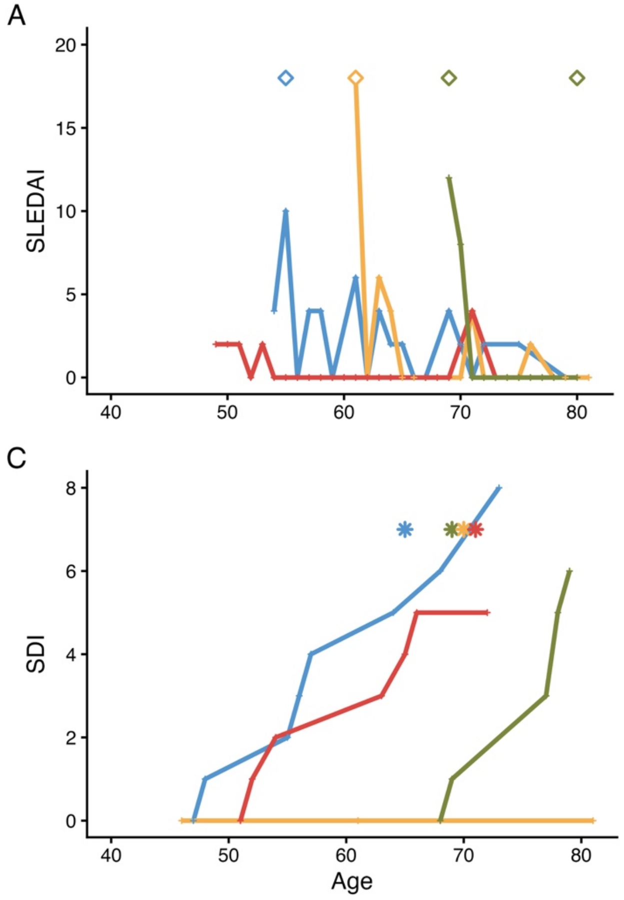

Supplementary figure S3. Longitudinal data from patients with neutralizing anti-IFN antibodies.

A) SLE Disease Activity Index, late-onset group, onset >65 years old. ◇=High-dose cortisone period**. B)** SLE Disease Activity Index, in early-onset group, onset <40 years old, n=5**. C)** SLICC Damage Index, late-onset group. ∗=Age at 1st anti-IFN. **D)** SLICC Damage Index, early-onset group. ∗=Age at 1st anti-IFN.
